# Solid-State NMR-Based Metabolomics Imprinting Elucidation in Tissue Metabolites, Metabolites Inhibition, and Metabolic Hub in Zebrafish by Chitosan

**DOI:** 10.3390/metabo12121263

**Published:** 2022-12-14

**Authors:** Raja Ganesan, Anirban Goutam Mukherjee, Abilash Valsala Gopalakrishnan, Vasantha-Srinivasan Prabhakaran

**Affiliations:** 1 Institute for Liver and Digestive Diseases, College of Medicine, Hallym University, Chuncheon 24253, Republic of Korea; 2 Department of Biological Sciences, Pusan National University, Busan 46241, Republic of Korea; 3 Department of Biomedical Sciences, School of Biosciences and Technology, Vellore Institute of Technology (VIT), Vellore 632014, India; 4 Department of Bioinformatics, Saveetha School of Engineering, Saveetha Institute of Medical and Technical Sciences (SIMATS), Chennai 602105, India

**Keywords:** zebrafish (*Danio rerio*), chitosan, metabolites, NMR spectroscopy, metabolomics, metabolic profiling, metabolic phenotyping

## Abstract

In this study, we demonstrated that chitosan-applied zebrafish (*Danio rerio*) tissue metabolite alteration, metabolic discrimination, and metabolic phenotypic expression occurred. The spectroscopy of solid-state ^1^H nuclear magnetic resonance (ss ^1^H-NMR) has been used. Chitosan has no, or low, toxicity and is a biocompatible biomaterial; however, the metabolite mechanisms underlying the biological effect of chitosan are poorly understood. The zebrafish is now one of the most popular ecotoxicology models. Zebrafish were exposed to chitosan concentrations of 0, 50, 100, 200, and 500 mg/L, and the body tissue was subjected to metabolites-targeted profiling. The zebrafish samples were measured via solvent-suppressed and T_2_-filtered methods with in vivo zebrafish metabolites. The metabolism of glutamate, glutamine, glutathione (GSH), taurine, trimethylamine (TMA), and its N-oxide (TMAO) is also significantly altered. Here, we report the quantification of metabolites and the biological application of chitosan. The metabolomics profile of chitosan in zebrafish has been detected, and the results indicated disturbed amino acid metabolism, the TCA cycle, and glycolysis. Our results demonstrate the potential of comparative metabolite profiling for discovering bioactive metabolites and they highlight the power of chitosan-applied chemical metabolomics to uncover new biological insights.

## 1. Introduction

Chitosan is a cationic polymer of (1–4)-linked 2-amino-2-deoxy-d-glucose interspersed with residual 2-acetamido-2-deoxy-d-glucose produced by deacetylation of chitin in basic conditions. Chitin is derived from the exoskeleton and skin of arthropods and insects and is the second most prevalent polysaccharide in nature after cellulose. It has been discovered that some bacteria, yeast, and fungi also produce chitin. The increasing use of chitosan in a wide range of pharmaceutical, biomedical, and biotechnological fields in recent years can be attributed to its mucoadhesion, low toxicity, biodegradability, and biocompatibility, as well as its antioxidant, antibacterial, antifungal, antitumor, and anti-inflammatory properties [1,2,3,4,5]. In its natural state, chitosan poses no threat to human health and is a viable material choice for medical applications. It has been shown that chitosan’s toxicity can be altered in either direction through chemical modification [6,7,8,9,10,11]. There was no evidence of toxicity in either terrestrial or aquatic animal studies [12,13,14] when the molecular weight of chitosan was reduced; nonetheless, one study demonstrated that acidic chitosan is harmful to cultured salmon, even if the chitosan’s properties and mode of action are still a mystery [15]. The hatching and survival rates of zebrafish embryos were decreased by medium-molecular-weight chitosan and CNPs, although the immune response was improved at low concentrations [16].

Metabolomics is the study of metabolites or small molecules found in a biofluid, using analytical chemistry techniques that are both exhaustive and non-selective. Metabolomics has significant promise for developing biomarkers, as metabolites represent the downstream activities of genes and proteins, and their levels may be assessed to learn more about the specific phenotype [17,18].

Zebrafish (*Danio rerio*) are freshwater fish. Due to their comfort of handling, fast growing feature, and quick transportation, zebrafish are ideally used for a wide range of scientific applications, such as in immunology [19], developmental biology [20], cancer biology [21], tissue regeneration [22], drug discovery [23], toxicity applications [24], genetic engineering [25], and bone-tissue engineering [26]. In this study, we investigated the regulation of targeted metabolites in zebrafish tissue by chitosan. To the best of our knowledge, there is no report in the literature on the use of chitosan-applied metabolomics, metabolic pathways, or metabolic discrimination in zebrafish tissue cellular metabolism. We provide strong evidence that metabolic phenotypes reflect chitosan-associated metabolites identified in screening experiments.

## 2. Materials and Methods

### 2.1. Chemicals and Reagents

Chitosan (product no.: 448869, CAS no.: 9012-76-4, deacetylation > 75%) was purchased from Sigma-Aldrich (St. Louis, MO, USA). The 3-(trimethylsilyl) propionic-2,2,3,3-d_4_ acid, sodium salt (TSP-d_4_) was purchased from Cambridge Isotope Laboratories, Inc., Tewksbury, MA 01876, USA. Deuterium water (D_2_O: 99.8% purity) was purchased from Cambridge Isotope Laboratories, United States. The extraction solvents of methanol (CH_3_OH), chloroform (CHCl_3_), sulfuric acid (H_2_SO_4_), and nitric acid (HNO_3_) were purchased from Carlo Erba Reagents, France. Flake fish food (TetraBits) was purchased from Tera (Mele, Germany). All other reagents were of HPLC grade.

### 2.2. Animal Maintenance

Zebrafish (*Danio rerio*) were acquired and acclimated for 14 days in 200 L glass aquarium with aerated dechlorinated water. We used male and female fish with an average age of 5 to 6 months. The mean weight and length of zebrafish are 0.4–0.8 g and 3.5–4.0 cm, respectively. Before the experiment, the photoperiod was maintained at 14 h light/10 h darkness at 27 ± 0.5 °C, and the pH of the water was maintained at 7.1–7.4. During acclimation, fish were fed 1% of their body weight twice daily with commercial food. All the animal experiments were performed by the Institutional Animal Care and Use Committee of Pusan National University (IACUC-21-0629), Korea.

### 2.3. Experimental Design and Standard Samples

According to previous reports [27], chitosan used in the range of 0, 50, 100, 200, and 500 mg/L was analyzed in zebrafish. For 15 min, a chitosan dispersed test solution was prepared using sonication. We calculated the body weight of the zebrafish. Following 14 days of acclimation, we examined several concentrations of chitosan (control, 0, 50, 100, 200, and 500 mg/L) for 72 h. Here, each experimental exposure contained 10 fish (*n* = 10, total 50) which were divided equally among all tanks. Tap water that had been dechlorinated with charcoal was used. The feeding was stopped. During the 0–72 h experiment, the water in each tank was replaced daily with fresh dechlorinated tap water containing the corresponding chitosan concentrations. After 72 h, all the zebrafish were euthanized in melting ice, and 10–25 mg of zebrafish body-tissue samples were isolated, then immersed in liquid nitrogen to stop enzymatic and chemical reactions, and stored at −80 °C for NMR analysis.

### 2.4. Sample Preparation for Solid-State ^1^H-NMR Analysis

Briefly, 20 mg wet weight of zebrafish tissue samples was directly dissolved in 25 μL of TSP-d_4_ (2.0 mM final concentration) and centrifuged appropriately. Next, each zebrafish sample was injected into a 4 mm zirconia MAS rotor (full volume: 40 μL) and covered with a Kel-F plastic cap. NMR tubes allowed the direct preparation of samples in NMR tubes. Sample preparation was completed within 5 min, and any bubbles or excess samples were removed. There was no loss in sample preparation or while transferring.

### 2.5. Solid-State NMR Spectroscopy Data Acquisition and Processing Parameters

An advanced method of magic-angle spinning (MAS: *θ* = 54.74°) high-resolution (HR) ^1^H-NMR was obtained, and the machine was equipped with a 14.1 T magnet (600 MHz ^1^H Larmore frequency), as described [28,29]. The solid-state NMR spectrometer (Bruker BioSpin Inc.) was maintained at 26 °C. The 3.2 mm MAS probes were used. The microwave (MW) irradiation was delivered to the NMR probe via corrugated waveguides about 4 m in total length. The solid-state electric-field-free probe with a diameter of 3.2 mm was used. To avoid excessive heating of the NMR samples, we set the power output of the gyrotron to 11 W (of a maximum 17 W). With the help of cold N_2_ gas, sample temperature was maintained, and a chiller unit supplied MAS driving and MAS bearing. The zebrafish sample was located in a sapphire rotor and stored in liquid N_2_ before the measurements were taken. The NMR probe was quickly cleaned with lint-free tissue to remove the condensation on the rotor walls. The sample catcher was inserted into the probe using a pressurized N_2_ gas line and connected to the chiller unit.

MAS ^1^H-NMR spectra were captured using a water-suppressed Carr–Purcell Meiboom–Gill-T_2_ pulse (CPMG-T_2_) spin-echo sequence (recycle delay: 90°-(−180°-)_n_-acquisition) [29]. NMR spectral shifts were measured concerning TSP-d_4_. The first and second pulses of spectral data acquisition were found at 216.0° and 108.0°, respectively. The spectral acquisition time and Fourier Transform (FT) size show 1.7 s and 32,768, respectively. Here, the 128 scans for each step were required to ensure a good signal-to-noise ratio. We covered the ^1^H-spectral resonance range from 0 to 20 ppm.

The spectral ^1^H-resonance width was found at 9615.4 Hz. At 0 ppm, TSP-d_4_ acts as a reference. To avoid statistical error, the water content sequence (δ 4.48–4.68 ppm) was removed. Finally, less than 10 min was needed for each sample. Parameters in these solid-state experiments were calibrated and optimized to ensure that the integration values of all spectra were precise. ssNMR is a powerful tool in metabolomics analysis.

### 2.6. Analytical Validation of Solid-State ^1^H-NMR, Data Handling, and Processing

The following basic parameters were considered and processed: standard binning length (δ 0.5–10.0 ppm) and bin size (0.001 ppm). The TSP-d_4_ spectrum (known concentration) was used as an internal reference to quantify other metabolites after spectrum acquisition, baseline correction, identification, quantification, and target profiling of metabolites present in the samples. All spectral data were processed with 0.5 Hz line broadening (lb) to attenuate the noise in the spectrum. Metabolites of ethanol and methanol were excluded from the analysis. The Excel file was obtained with targeted metabolite concentrations (mM). The metabolites were identified and quantified based on accurate mass, secondary fragmentations, and HMDB (https://hmdb.ca/) (accessed on 1 July 2022).

### 2.7. Validation of Metabolic Pathways

All binned NMR values were widely exported to MetaboAnalyst software (version 5.0; https://www.metaboanalyst.ca/) (accessed on 1 July 2022). Score plot analysis, such as unsupervised principal component analysis (PCA), supervised particle least squares discriminant analysis (PLS-DA), supervised orthogonal PLS-DA (OPLS-DA), variable importance in projection (VIP > 1), and heatmap correlation, were applied to NMR data. Among all the score plots, every dot represented ^1^H-NMR data. The goodness of fit (R^2^) and goodness of prediction (Q^2^) were calculated from the score plots. Using the *Pareto scaling* algorithm, the data normalization, batch correction, and validation were evaluated, which delivered the variables according to the square root of their standard deviations (SD). Metabolomics pathway analysis (MetPa) was done via the global test algorithm for pathway enrichment and relative centrality to assess the metabolites’ importance.

### 2.8. Statistical Analysis

To find all variables, Pearson’s correlation coefficient, outliers, missing data detections, normalization, a *t*-test, an ANOVA test (*p*-value 0.05), and fold change (FC: >1.0% increased and FC: <1.0% decreased) were performed to assess the significant differences and biochemical parameters among the samples. The metabolic changes were considered significant if the *p*-value was less than 0.05 and correlated using the Holm–Bonferroni method. Pathways were considered significant if the Holm p was less than 0.05, the false discovery rate (FDR) was less than 0.05, and the impact value was greater than 0. GraphPad Prism 8 software was used to plot the graphs. The data values were expressed as the average mean ± SD.

## 3. Results

### 3.1. High-Cover Metabolomics of Chitosan in Zebrafish

**Figure 1:**
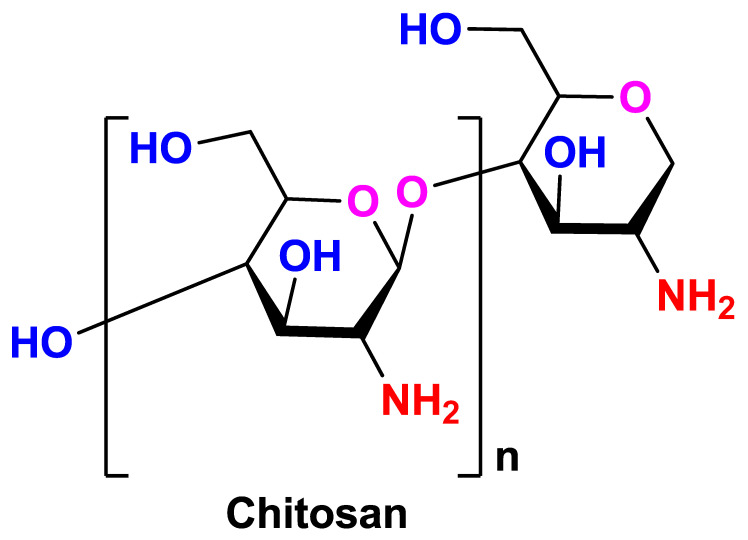
The chemical structure of solid chitosan.

Figure 1 depicts the schematic structure of chitosan. D-glucosamine and N-acetyl-D-glucosamine are joined together to form the polysaccharide known as chitosan. It is created by deacetylating chitin, a carbohydrate found in nature.

**Figure 2:**
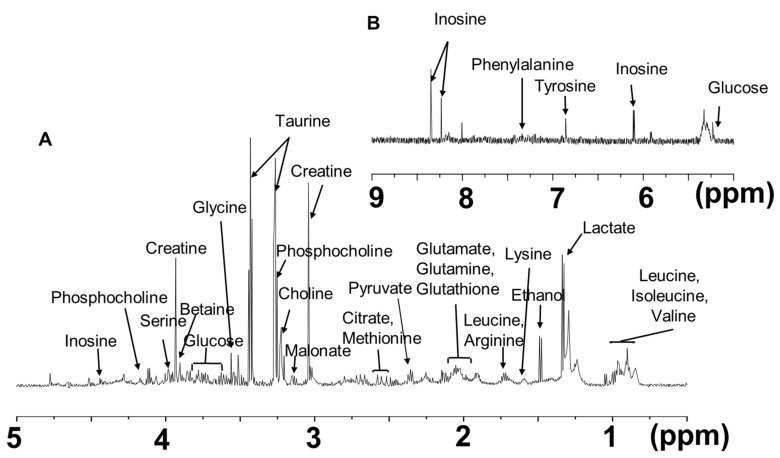
Chemical structure of chitosan. ^1^H-NMR spectra (MAS: *θ* = 54.74°) of chitosan samples in zebrafish. Spectral aliphatic and aromatic regions of interest recorded on a model mixture of samples. (**A**) typical 600 MHz ^1^H-NMR spectra (δ 0–5 ppm: aliphatic region) of zebrafish tissue. (**B**) an aromatic region (δ 5–9 ppm) of ^1^H-NMR experiments in zebrafish tissue.

Using ^1^H-NMR, we detected and aligned spectral features into master peak tables for each sample type, providing total targeted metabolites that ranged from 0–9 ppm in zebrafish. An apliphatic region (δ 0–5 ppm) and an aromatic region (δ 5–9 ppm) were assessed to screen for spectral analysis (Figure 2A,B). A cheminformatic workflow with focused annotation techniques was used to gather ^1^H-NMR spectra for all these features and to achieve the maximum metabolome coverage achievable. The focused technique matched the known metabolites with 50 literature reports of microbial by-products, including neurotransmitters, osmotic regulators, energy producers, etc., and an internal spectral library with integration, accurate resolution, and proton characteristic fragments of metabolites. The targted metabolites are reported in Table 1.

### 3.2. Metabolic Discrimination of Chitosan Groups in Zebrafish

**Figure 3:**
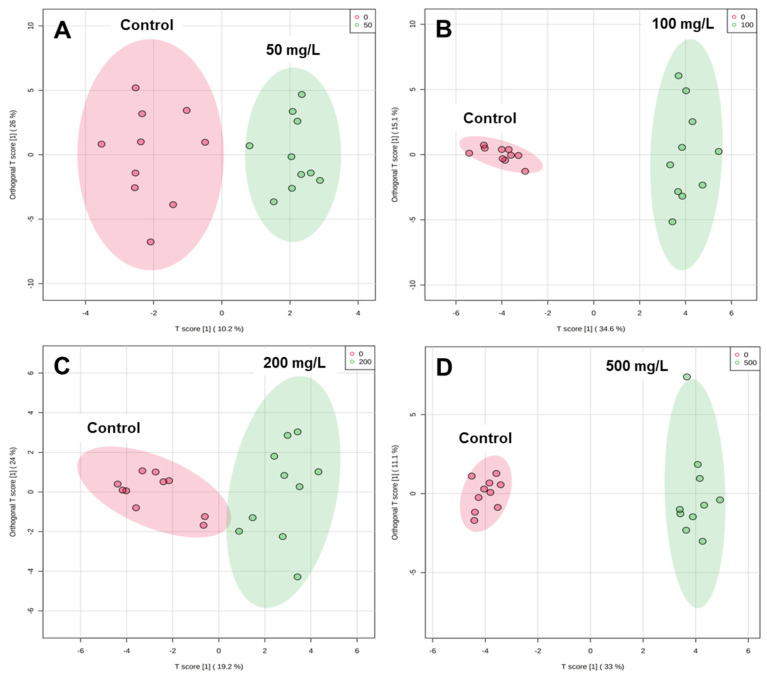
A plot of OPLS-DA scores in fish-tissue samples with four studied groups and control samples: control (red), chitosan (green): (**A**) 0 vs. 50 mg/L. PC has a total metabolic difference of 36.2%; (**B**) 0 vs. 100 mg/L. PC has a total metabolic difference of 49.7%; (**C**) 0 vs. 200 mg/L. PC has a total metabolic difference of 43.2%; (**D**) 0 vs. 500 mg/L. PC has a total metabolic difference of 44.1%.

The majority of the variance is accounted for in the principal components (PC). There is a good metabolic differentiation between control and chitosan concentration (Figure 3). The 50, 100, 200, and 500 mg/L of chitosan groups are clearly separated from the untreated control group by the OPLS-DA score plot in Figure 3A–D, suggesting that the metabolic changes are too minor to be detected by an supervised multivariate technique. The OPLS-DA score plot shows better metabolic variance in 36.2% (50 mg/L), 49.7% (100 mg/L), 38.2% (200 mg/L), and 44.1% (500 mg/L). The 49.7% of metabolic difference was found more in 100 mg/L of chitosan than others. PC hold a significant amount of variance. The samples including the control and 50, 100, 200, and 500 mg/L of chitosan samples were included in the initial evaluation of the analytical system’s overall performance using PCA analysis (Figure S1).

This metabolic difference of chitosan treatment was further confirmed with a 100-premutation test, which could verify its stability and reliability. The OPLS-DA score plot in this case did not over-fit (Figure 3).

### 3.3. Exploration and Discrimination of Metabolites in Zebrafish Tissue

**Figure 4:**
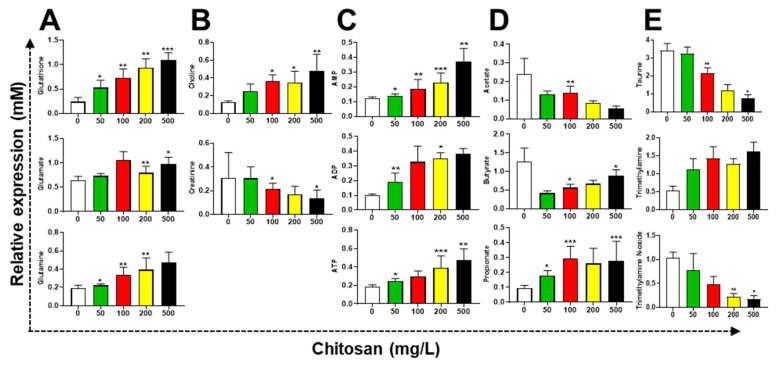
Metabolites altered in zebrafish tissue cells treated for 72 h with chitosan treatment. (**A**) Glutathione, glutamate, and glutamine. (**B**) Choline, and creatinine. (**C**) AMP, ADP, and ATP, (**D**) SCFAs: acetate, butyrate, and propionate. (**E**) Taurine, trimethylamine N-oxide, and trimethylamine. The zebrafish metabolites were significantly increased and decreased by chitosan. The data are expressed as the mean ± standard deviation (*n* = 10). *, ** and *** represent *p* < 0.05, *p* < 0.01, and *p* < 0.001, respectively.

The levels of glutathione, glutamate, and glutamine were increased in zebrafish tissues. The levels of choline and creatinine were increased and decreased in zebrafish tissues, respectively (Figure 4A,B). Glutathione is one of the main cellular antioxidants, which could be altered in cellular wellness. This evidence suggests that cells were not dead, but they may have been compromised. The bacterial iron-sparing response may be connected to the rise in fermentation metabolites [30]. The iron-dependent enzyme aconitase was used in the process of converting the TCA cycle intermediate ketoglutarate into glutamine and glutamate. The chitosan treatment increased AMP, ADP, and ATP levels. In the zebrafish gut, the short-chain fatty acids (SCFAs: acetate, butyrate, and propionate) were altered. Chitosan concentration degraded the acetate and butyrate in fish tissue. Surprisingly, propionate levels were significantly higher than those in the control group (Figure 4C,D).

Different pathways can be used in the gut to produce these SCFAs. First, propionate and butyrate directly inhibit histone deacetylases to regulate gene expression (HDACs). By regulating the integrity of the gut mucosal epithelial barrier and enhancing metabolic balance, SCFAs promote liver regeneration [31]. It has been demonstrated that SCFAs increase body weight by promoting the expression of GPR41 and GRP109A. Acetate, propionate, and butyrate may bind to GPR43 and GPR41, whose amino acid sequences are 43% homologous [32]. Interestingly, the down-regulated taurine and trimethylamine N-oxide (TMAO) by chitosan treatment were primarly associated with osmolytic function. The trimethylamine (TMA) was increased in tissues by chitosan (Figure 4E). The osmolytes, such as taurine, trimethylamine, and TMAO, had changed, indicating that the global metabolites had started to adjust to oxidative stress. The osmoregulation mechanism was exacerbated to decrease betaine levels when the dosage of chitosan was raised [33].

### 3.4. Metabolic Parthways Analysis

**Figure 5:**
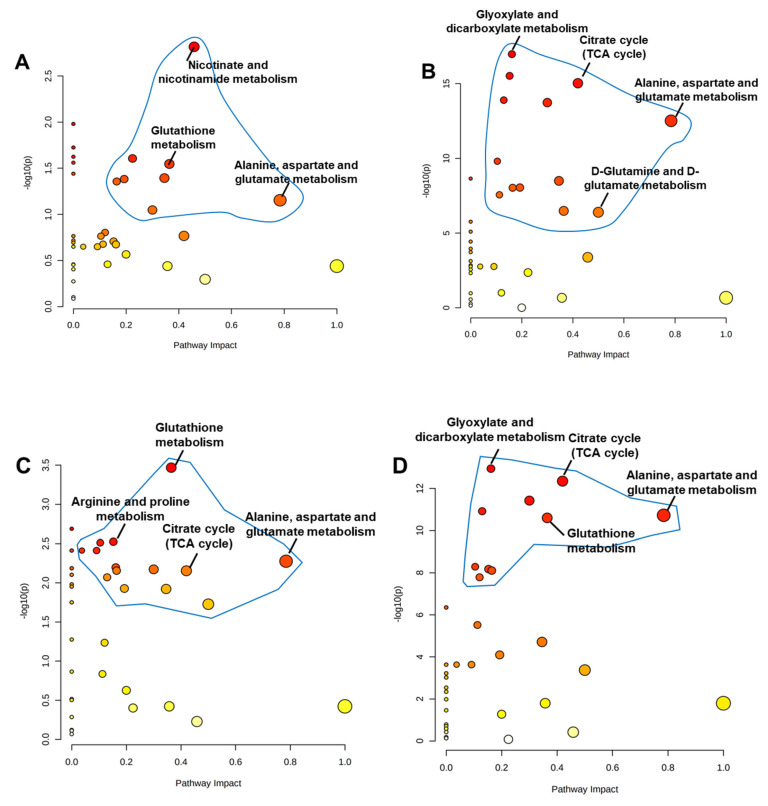
Chitosan was treated in experimental groups of (**A**) 50 mg/L, (**B**) 100 mg/L, (**C**) 200 mg/L, and (**D**) 500 mg/L in zebrafish. Each bubble in the images represents an increased metabolic pathway impact in zebrafish tissues. According to topological analysis (TA), the size and abscissa of the bubble represent the strength of the metabolic pathway’s impact; the bigger the bubble, the more significant the influence. The ordinate and the color intensity of the bubbles show the significant p value of enriched metabolic pathways. The metabolites with an adjusted *p*-value < 0.05 and VIP > 1 were considered significantly different.

Changes in the metabolomic profiles and metabolic pathways were examined under various chitoson concentrations. The examination of the volcanic map revealed that the metabolites in the intestines of chitosan-fed 50, 100, 200, and 500 mg/L of chitosan were all differentially expressed (Figure 5A–D). The alanine, aspartate, and glutamate metabolisms, glutathione metabolism, and nicotinate and nicotinamide metabolisms were significantly altered in zebrafish tissue pathways after 50 mg/L chitosan treatment (Figure 5A). D-glutamine and D-glutamate metabolism, alanine, aspartate, and glutamate metabolism, the TCA cycle, and glyoxylate and dicarboxylate metabolism were all significantly altered by 100 mg/L chitosan treatment (Figure 5B). In 200 mg/L of chitosan treatment, the alanine, aspartate, and glutamate metabolisms, the TCA cycle, the arginine and proline metabolisms, and glutathione metabolism were all significantly affected (Figure 5C). Finally, the alanine, aspartate, and glutamate metabolisms, the TCA cycle, glutathione metabolism, and the glyoxylate and dicarboxylate metabolisms were inhibited in fish with 500 mg/L of chitosan treatment (Figure 5D).

## 4. Discussion

We have demonstrated that the quantification of metabolites in zebrafish tissues is influenced by the presence of chitosan using pure shift ^1^H-NMR spectra recorded with water-signal suppression. A robust analytical protocol based on a library of reference pure shift spectra can be implemented to determine metabolite concentration by fitting their spectrum with a reference signal, which allows accounting for small spectrometer and pulse sequence instabilities. This is made possible by the high reproducibility and quality of the data that can be obtained using state-of-the-art ultra-high-resolution methods. We verified our methodology using tissue samples.

It was previously observed that chitosan is hazardous to zebrafish embryos and that the size, buffer, and incubation duration may have an impact on the mortality [27]. An earlier study examined the toxicity of chitosan.

In our analysis, zebrafish exposed to chitosan increased their glutathione metabolism; as a result, chitosan exposure causes oxidative stress in the cellular metabolism of fish tissue. The variations in AMP, ADP, and ATP levels indicate problems with glycolysis/gluconeogenesis imbalance, which highlight the possibility of fish liver injury following chitosan exposure. Tissue SCFA concentrations vary throughout the chitosan treatment in zebrafish. TMA–TMAO collaboration highlights the importance of these metabolites in a variety of biological processes. As an osmolyte, TMAO can prevent cellular proteins from being denaturized by urea and other disruptive osmolytes. It can also protect invertebrates and other marine animals from osmotic stress, cold temperatures, and high hydrostatic pressure.

Many altered metabolic pathways of amino acid metabolism, including the production of valine, leucine, and isoleucine as well as the metabolism of glycine, serine, and threonine, were seen in zebrafish exposed to chitosan. Zebrafish treated with chitosan had aberrant alanine, aspartate, and glutamate metabolism, and TCA cycle biosynthesis pathways. The aberrant metabolic pathway and glycolysis have a close relationship. Zebrafish exposed to chitosan appeared to have altered energy metabolism, as seen by the abnormal metabolic pathways of glycolysis, the TCA cycle, and glyoxylate and dicarboxylate metabolism. Muscle breakdown may also be connected to protein oxidative damage since the pathways associated with amino acids were up-regulated, primarily in the larvae exposed to chitosan; as a result, the growth rate was slowed in a way that depended on chitosan concentration [34].

Zebrafish exposed to chitosan showed an increase in the metabolism of choline, which includes phosphocholine and glycerophosphocholine. There is an alteration process between the metabolites choline, phosphocholine, and glycerophosphocholine caused by chitosan treatment. Choline is an osmoprotectant as well; however, it is less efficient than glycine betaine [35]. This study examined how oral exposure to chitosan in zebrafish affected their metabolism. The risk assessment of chitosan metabolites has been widely reported.

## 5. Conclusions

In this study, we report the quantification of metabolites in zebrafish tissues and provide the metabolic alterations by chitosan. We used ssNMR to examine the metabolism of chitosan in zebrafish. Metabolomics profiling was performed on zebrafish exposed to chitosan. Samples of zebrafish metabolites in their natural environment were analyzed using solvent-suppressed and T_2_-filtered techniques. Changes were also observed in the metabolites of AMP, ADP, ATP, glutamate, glutamine, glutathione, choline, taurine, TMA, TMAO, and SCFAs. There was a change in the metabolomics profile, and the data showed that amino acid metabolism, the SCFAs, TCA cycle, and glycolysis were all disrupted. Our findings demonstrate the utility of chitosan-applied chemical metabolomics in the search for novel biological insights and the promise of comparative metabolite profiling in the search for bioactive metabolites.

## Figures and Tables

**Table 1:** Solid-state NMR spectral data setting, splitting of metabolites, and targeting and quantification of chitosan-treated cellular metabolites.

KEGG	HMDB	Metabolites	Hit	PubChem	Chemical Formula	MW (Da)	δ ^1^H (ppm) and Multiplicity
C00033	HMDB0000042	Acetate	Acetic acid	176	C_2_H_4_O_2_	60.05	1.90 (s)
C00049	HMDB0000191	Aspartate	L-Aspartic acid	5960	C_4_H_7_NO_4_	133.10	3.89 (dd); 2.80 (dd); 2.66 (dd)
C00041	HMDB0000161	Alanine	L-Alanine	5950	C_3_H_7_NO_2_	89.09	3.78 (q); 1.47 (d)
C00152	HMDB0000168	Asparagine	L-Asparagine	6267	C_6_H_14_N_4_O_2_	132.12	3.76 (t); 1.90 (m);1.68 (m)
C00002	HMDB0000538	ATP	Adenosine triphosphate	5957	C_10_H_16_N_5_O_13_P_3_	507.18	8.52 (s); 8.12 (d); 4.50 (m); 4.21 (m)
C00008	HMDB0001341	ADP	ADP	6022	C_10_H_15_N_5_O_10_P_2_	427.201	8.54 (s); 5.94 (m); 4.11 (m); 4.00 (m)
C00020	HMDB0000045	AMP	Adenosine monophosphate	6083	C_10_H_14_N_5_O_7_P	347.221	8.22 (s); 6.16 (s); 4.53 (dd); 4.34 (d)
C00719	HMDB0000043	Betaine	Betaine	247	C_24_H_26_N_2_O_13_	550.45	3.89 (s); 3.25 (s)
C00158	HMDB0000094	Citrate	Citric acid	311	C_6_H_8_O_7_	192.12	2.65 (d); 2.53 (d)
C00114	HMDB0000097	Choline	Choline	305	C_5_H_14_NO	104.17	4.05 (dd), 3.50 (dd),3.18 (s)
C00122	HMDB0000134	Fumarate	Fumaric acid	444,972	C_4_H_4_O_4_	116.072	6.51 (s)
C00221	HMDB0000122	Glucose	D-Glucose	5793	C_6_H_12_O_6_	180.16	5.22 (d); 4.64 (d); 3.88 (dd); 3.72 (m); 3.40 (m); 3.39 (m); 3.21 (dd)
C00051	HMDB0062697	Glutathione	Glutathione	745	C_10_H_17_N_3_O_6_S	307.32	4.20 (q); 3.78 (m); 2.97 (dd); 2.15 (m)
C00025	HMDB0000148	Glutamate	L-Glutamic acid	33,032	C_5_H_9_NO_4_	147.129	3.76 (t); 2.44 (m); 2.12 (m)
C00064	HMDB0000641	Glutamine	L-Glutamine	5961	C_5_H_10_N_2_O_3_	146.144	5.75 (m); 7.80 (m); 6.16 (t)
C00186	HMDB0000190	Lactate	L-Lactic acid	107,689	C_3_H_6_O_3_	342.3	4.10 (q); 1.32 (d)
C00407	HMDB0000172	Isoleucine	L-Isoleucine	6306	C_6_H_13_NO_2_	131.17	3.66 (d); 1.96 (m); 0.99 (d); 0.92 (t)
C00183	HMDB0000883	Valine	L-Valine	6287	C_5_H_11_NO_2_	117.146	3.60 (d); 2.261 (m); 0.97 (d)
C00123	HMDB0000687	Leucine	L-Leucine	6106	C_6_H_13_NO_2_	131.17	3.72 (m); 1.70 (m); 0.94 (t)
C00294	HMDB0000195	Inosine	Inosine	6021	C_10_H_12_N_4_O_5_	268.23	8.30 (s); 6.05 (d); 4.42 (dd); 3.82 (dd)
C00036	HMDB0000223	Oxalacetate	Oxalacetic acid	970	C_4_H_4_O_5_	132.071	2.37 (s)
C08645	HMDB0001844	Methylsuccinate	Methylsuccinic acid	10,349	C_5_H_8_O_4_	132.11	2.61 (m); 2.51 (dd); 2.11 (dd); 1.07 (d)
C00137	HMDB0000211	Myo-Inositol	myo-Inositol	-	C_6_H_12_O_6_	180.16	4.05 (t); 3.61 (t); 3.52 (dd); 3.26 (t)
C00079	HMDB0000159	Phenylalanine	L-Phenylalanine	6140	C_9_H_11_NO_2_	165.19	7.33 (d); 7.37 (m); 7.43 (m)
C00022	HMDB0000243	Pyruvate	Pyruvic acid	1060	C_3_H_4_O_3_	88.0621	2.46 (s)
C00042	HMDB0000254	Succinate	Succinic acid	1110	C_4_H_6_O_4_	118.088	2.39 (s)
C00245	HMDB0000251	Taurine	Taurine	1123	C_2_H_7_NO_3_S	125.15	3.43 (t); 3.42 (t); 3.25 (t)
C00188	HMDB0000167	Threonine	L-Threonine	6288	C_4_H_9_NO_3_	119.12	4.24 (m); 3.57 (d); 1.31 (d)
C00078	HMDB0000929	Tryptophan	L-Tryptophan	6305	C_11_H_12_N_2_O_2_	204.26	7.72 (d); 7.31 (s); 4.04 (dd); 3.29 (dd)
C00565	HMDB0000906	Trimethylamine	Trimethylamine	1146	C_3_H_9_N	59.1103	2.89 (s)
C00082	HMDB0000158	Tyrosine	L-Tyrosine	6057	C_9_H_11_NO_3_	181.188	7.24 (d); 6.94 (m); 3.34 (dd); 3.30 (dd)
C01104	HMDB0000925	TMAO	Trimethylamine N-oxide	1145	C_3_H_9_NO	75.11	3.25 (s)
C00385	HMDB0000292	Xanthine	Xanthine	1188	C_5_H_4_N_4_O_2_	152.110	7.892 (s)

Note and abbreviations: singlet (s); doublet (d); triplet (t); doublet of doublets (dd); quartet (q); multiplets (m); molecular weight (MW); parts-per-million (ppm); adenosine monophosphate (AMP); adenosine diphosphate (ADP); adenosine triphosphate (ATP); and Trimethylamine N-oxide (TMAO).

## Data Availability

The data presented in this study are available in the article.

## Supplementary Materials

The following supporting information can be downloaded at: https://www.mdpi.com/article/10.3390/metabo12121263/s1, Figure S1: PCA with five principal components models generated from the NMR metabolomics data set.
