# Supplementary material for: Solid-State NMR-Based Metabolomics Imprinting Elucidation in Tissue Metabolites, Metabolites Inhibition, and Metabolic Hub in Zebrafish by Chitosan
Source: Metabolites. 2022 Dec 14;12(12):1263. doi: 10.3390/metabo12121263 (PMC9785866; doi:10.3390/metabo12121263)
Supplement: Supplementary file 1 [file metabolites-12-01263-s001.zip › metabolites-2074133-supplementary.pdf]

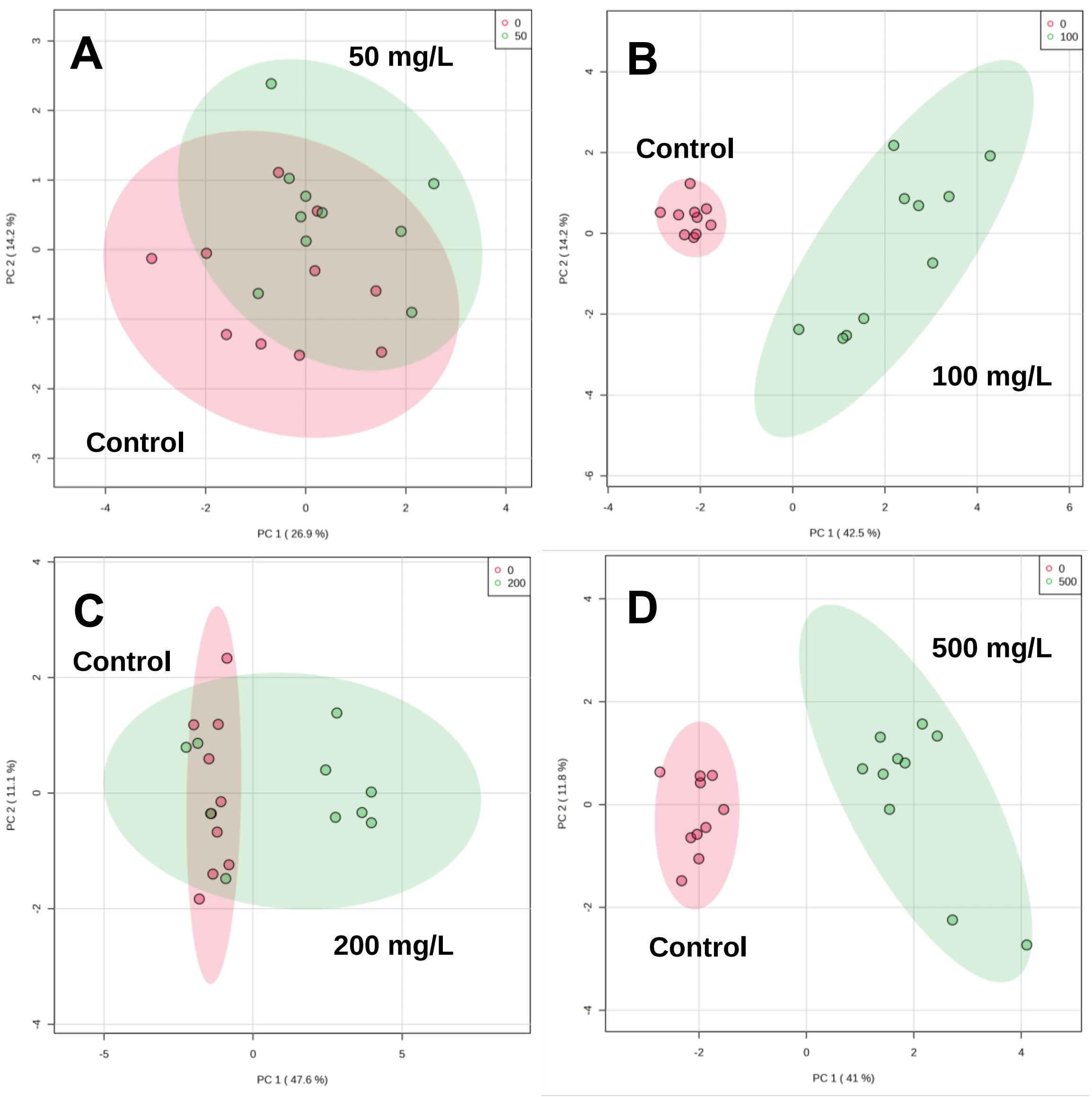

Figure S1. PCA with five principal components models generated from the NMR metabolomics data set. Zebrafish were treated with chitosan (green dot) and control (red dot) Ellipses represent a 95% confidence limit of the normal distribution of each cluster.
